# Effects of co-application chemical and organic fertilizers on microbial residues and their contribution to soil organic carbon accumulation

**DOI:** 10.3389/fmicb.2026.1819222

**Published:** 2026-05-08

**Authors:** Zhimei Yang, Zehui Wei, Hongrui Li, Yi Chen, Khanom Simarani, Yonglei Jiang

**Affiliations:** 1 Yunnan Academy of Tobacco Agricultural Sciences, Kunming, China; 2 Biotechnology and Applied Microbiology Laboratory, Division of Microbiology, Institute of Biological Sciences, Faculty of Science, Universiti Malaya, Kuala Lumpur, Malaysia

**Keywords:** amino sugars, fertilization strategies, microbial residues, organic fertilizer, soil organic carbon

## Abstract

**Background:**

Fertilization strategies in agricultural ecosystems significantly influence soil organic carbon (SOC) dynamics, and integrated chemical and organic fertilization may enhance short-term SOC accumulation. However, the mechanisms, particularly the contribution of microbial residues to SOC dynamics during the crop growth period, remain unclear.

**Methods:**

In this study, we evaluated four fertilization treatments in tobacco-planting field under a randomized block design with three blocks: (1) no fertilizer (CK), (2) chemical fertilizer (CF), (3) chemical fertilizer with corn straw amendment (CFS), and (4) chemical fertilizer with manure amendment (CFM). Soil samples (0–20 cm) were collected at six stages of the tobacco-growing period, and were analyzed for SOC, soil physicochemical properties, and amino sugar biomarkers (MurA, GalN, and GluN) to calculate microbial residue-C fractions.

**Results:**

Fertilization significantly affected soil nutrient status, amino sugar pools, microbial residue-C fractions, and SOC over time compared with CK and CF, the two organic-amended treatments were associated with higher SOC concentrations, and CFM showed the strongest short-term SOC response (peaked at 10.30 ± 0.22 g kg^−1^ at 30 d). Amino sugar concentrations and microbial residue-C fractions were generally higher under CFM and CFS than under CF and CK, especially during the middle and late stages. Correlation analysis showed that SOC was positively correlated with BRC and MurA under CFM (both *r* = 0.58, *P* < 0.05). The merged piecewise linear mixed-effects SEM indicated that fertilization treatment, time, and their interaction all had significant positive effects on soil physicochemical properties, microbial residues, and SOC.

**Conclusion:**

Integrated chemical-organic fertilization, particularly CFM, favored short-term SOC accumulation in tobacco-planting soil. Amino sugar-derived microbial residues were also enhanced under organic-amended fertilization, and bacterial residues were more closely associated with SOC than fungal residues during the observed period. The merged piecewise linear mixed-effects SEM indicated that short-term SOC dynamics were driven mainly by fertilization regime and temporal variation, providing region-specific evidence for optimizing fertilization strategies in tobacco-planting red soils of Yunnan.

## 1 Introduction

The soil organic carbon (SOC) pool represents the largest carbon reservoir within terrestrial ecosystems (Raza et al., 2024). Globally, the SOC stock is estimated to be approximately three times larger than either the atmospheric or terrestrial vegetation carbon pool (Lehmann and Kleber, 2015). As a fundamental indicator of soil health, SOC dynamics not only influence the sustainability of agricultural ecosystems but also have far-reaching implications for the stability of the atmosphere and biosphere (Stockmann et al., 2013; Scharlemann et al., 2014). However, unsustainable fertilization practices, such as excessive application of chemical fertilizers, neglect of organic matter amendments, and continuous monocropping—have led to significant declines in soil fertility and SOC depletion. These adverse effects manifest in the form of carbon pool degradation, soil structural deterioration, and microbial imbalance, ultimately imposing severe constraints on sustainable agricultural development (Guillaume et al., 2015; Li et al., 2020b). Therefore, the implementation of scientifically sound and effective agricultural management practices is crucial for enhancing soil health and mitigating short-term SOC loss.

Soil microbial communities serve as critical regulators of SOC formation and decomposition through their dual functional roles in biogeochemical cycling (Trivedi et al., 2013). Although microbial biomass accounts for only 1 %−5 % of total SOC, it governs essential soil processes including organic matter decomposition, humification, and nutrient transformation. Microbes assimilate exogenous carbon, primarily plant-derived carbon, through anabolic metabolism and transform it into microbial residues, thereby contributing to SOC formation and retention (Liang et al., 2020; Wang et al., 2021). Microbial residues have emerged as a critical biomarker for evaluating the microbial contribution to SOC accumulation and are increasingly recognized for their essential role in soil carbon stabilization and cycling (Liang et al., 2017; Liu et al., 2019). Notably, empirical evidence reveals that microbial residues are particularly prone to stabilization through associations with silt and clay minerals (Cotrufo and Lavallee, 2022), making them an important indicator of long-term agricultural management effects, particularly in conservation-oriented practices such as crop residue retention (Cotrufo and Lavallee, 2022).

Amino sugars, as major structural components of microbial cell walls, are known to persist in soils for extended periods following microbial cell death (Joergensen, 2018). Amino sugars are widely recognized as specific biomarkers of microbial residues in soils (Ni et al., 2020), and numerous studies have employed them to evaluate the dynamics of microbial necromass carbon and its contribution to SOC (Yang et al., 2022; Li et al., 2023; Salas et al., 2024). Current quantification methods primarily focus on four key biomarkers: glucosamine (GluN), galactosamine (GalN), muramic acid (MurA), and mannosamine (ManN) (Zhang and Amelung, 1996). These compounds exhibit distinct microbial origins, GluN predominantly derives from fungal chitin in cell walls, while MurA serves as a bacterial-specific indicator through its exclusive presence in peptidoglycan of bacterial cell walls (Glaser et al., 2004; Appuhn and Joergensen, 2006). Analyzing amino sugar biomarkers provides critical insight into the fate of microbial communities within organic matter and helps clarify the role of microbial residues in SOC dynamics (van Groenigen et al., 2010). Furthermore, study demonstrated climate-dependent enhancement of microbial necromass contributions to SOC accumulation via metabolic pathway modulation (Ni et al., 2021), and amino sugars have also been shown to reflect the impacts of agricultural management practices on soil microbial communities (Ding et al., 2011).

Fertilization practices exert critical regulatory control over SOC dynamics and microbial community structure in agroecosystems by altering soil microecological conditions (Wang et al., 2024). The prevalent intensive agricultural model, characterized by excessive chemical fertilizer application, has led to serious soil degradation including nutrient imbalances, reduced microbial diversity, and substantial SOC loss (Guo et al., 2010; Cai et al., 2015; Tkaczyk et al., 2020; Liu et al., 2024). In response partial substitution of chemical fertilizers with organic amendments not only maintains crop yields but also significantly enhances short-term SOC enrichment (Luo et al., 2018), stimulates microbial biomass and metabolic activity, and improves soil physical properties, representing a key strategy for achieving reduced fertilizer input with increased efficiency (Liu et al., 2023). Nevertheless, in tobacco-planting soils, the mechanisms underlying amino sugar accumulation in response to different fertilization patterns remain poorly understood. Elucidating this phenomenon is essential for uncovering the mechanisms governing SOC enrichment in region-specific agricultural systems—particularly in tobacco-dominated agricultural systems, and for optimizing short-term carbon accumulation strategies in these contexts.

Tobacco (*Nicotiana tabacum* L.), as a major leaf-type cash crop, plays a pivotal role in both agricultural and fiscal systems, particularly in China where it occupied 1.565 million hectares (1.05 × 10^6^ ha) of cultivated land in 2022 (State Tobacco Monopoly Administration, 2023; State Administration of Taxation, 2023), according to the (State Tobacco Monopoly Administration 2023). Among all provinces, Yunnan contributes around 40% of the national flue-cured tobacco production, with the tobacco industry serving not only as a stable source of income for local farmers but also as a key driver of regional economic development. However, under escalating land constraints and profit-driven intensification, 30–60 % of tobacco fields practice continuous cropping systems (Li et al., 2024), which has led to severe degradation of soil physicochemical properties, nutrient imbalances, and pathogenic fungi accumulation. In recent years, scientifically-informed fertilization practices have been recognized as effective strategies to alleviate the adverse effects of continuous monoculture, improve soil microecological conditions, and enhance both yield and quality of tobacco (Wang et al., 2022b). The combined application of organic and inorganic fertilizers has been found to improve SOC stability (Xu et al., 2024). The emergence of novel biofertilizers and microbial function-based regulation strategies offers promising approaches for managing the challenges of continuous tobacco cropping (Wang et al., 2022c). However, the underlying mechanisms of short-term SOC accumulation under different fertilization treatments in tobacco-planting soils remain insufficiently understood. Therefore, identifying scientifically fertilization strategies that promote nutrient balance, reshape microbial community structure, and enhance soil carbon enrichment is essential for ensuring the long-term sustainability of tobacco production.

Here, based on a field experiment conducted at the Yanhe monitoring site in a tobacco-planting region of Yunnan Province, China, we aimed to (1) investigate the effects of different fertilization treatments on soil physicochemical properties and microbial residue carbon dynamics during the flue-cured tobacco growth period, and (2) clarify the microbial mechanisms driving SOC accumulation under various fertilization regimes. We hypothesized that: (1) organic amendments (straw and manure) would enhance microbial-derived carbon accumulation compared to CK and CF, due to increased substrate availability and microbial turnover, and (2) manure would result in a higher microbial necromass contribution than straw, owing to its balanced nutrient composition and stronger stimulation of microbial growth. This study aims to elucidate how different fertilization strategies influence SOC dynamics via microbial pathways, providing insights into sustainable fertilization practices that enhance soil health and promote short-term SOC enrichment in tobacco-growing soils.

## 2 Materials and methods

### 2.1 Site description

This study was conducted in Yanhe Research Farm, Yunnan Academy of Tobacco Agricultural Sciences near Yuxi, Yunnan, China (24°14' N, 102°30' E), where altitude is 1680 m with an annual precipitation and mean temperature of 918 mm and 27 °C, respectively (Li et al., 2019). Within the same platform, randomized complete-block experiments were conducted in 2016 comparing tobacco monocropping vs. tobacco–rice rotation and N fertilizer rates (0, 75, 112 kg N ha^−1^), with an additional treatment of 60 kg N ha^−1^ plus 15,000 kg ha^−1^ humified swine compost. According to platform management records, the long-term field platform completed 18 seasons of flue-cured tobacco monocropping and 9 years of tobacco–rice rotations during 1998–2016 (cultivar K326), all tobacco residues (including roots) were typically removed in tobacco seasons. In contrast, in rice seasons ~2,000 kg ha^−1^ of residues were retained, and N fertilizer of about 240 kg N ha^−1^ was commonly applied. For the present work, the target plot had been under continuous flue-cured tobacco cultivation from 2004 to 2021 (18 consecutive seasons) prior to our sampling. Apart from those specific historical trial seasons, routine management of this plot did not include intentional, large-scale organic amendments, crop residues followed local practice. The baseline soil properties were recorded with pH of 6.4, soil organic matter of 10.70 g kg^−1^, total nitrogen of 0.54 g kg^−1^, total phosphorus of 0.11 g kg^−1^, total potassium of 6.43 g kg^−1^, available nitrogen of 82.0 mg kg^−1^, available phosphorus of 90.0 mg kg^−1^, available potassium of 160.0 mg kg^−1^ (Zou et al., 2018). The initial soil texture, determined by the hydrometer method, consisted of 28% sand, 50% silt, and 22% clay (Gee and Or, 2002).

### 2.2 Experimental design

In 2022, the experiment was carried out in a randomized block design with three blocks. Within each block, the four fertilization treatments were randonmly assigned to four plots, each treatment occurred once per block. Each plot measured 28 m^2^ (14 m × 2 m) and remained a fixed experimental unit throughout the study. Four treatments were: (1) no fertilizer application (CK), (2) chemical fertilizer alone (CF), (3) chemical fertilizer with corn straw (CFS), and (4) chemical fertilizer with manure (CFM). Chemical fertilizer (N: P_2_O_5_: K_2_O = 1:1:3) was split into three applications: 50 % applied as a basal dose, 25% top-dressed at 7 d after transplanting, and the remaining 25% top-dressed at 20 d after transplanting. Chemical fertilizer application rates were 112 kg ha^−1^ in CF, 60 kg ha^−1^ in both CFS and CFM, with the corresponding nutrient inputs summarized in Table 1. The properties of corn straw and pig manure were analyzed by Yunnan Sanbiao Agricultural and Forestry Technology Co., Ltd. and are shown in Table 2. To match nominal total nitrogen inputs among fertilized treatments, we topped up the N deficit in CFS and CFM (relative to CF) using the measured total N of corn straw 6.8 g N kg^−1^ and manure 59.43 g N kg^−1^ (Table 2). No mineralization coefficient was applied (short-term field trial, conservative assumption). Let R be the chemical fertilizer rate (kg ha^−1^), and *W*_*N*_, *W*_*P*_2_*O*_5__, *W*_*K*_2_*O*_ be mass fractions (0.20, 0.20, 0.60). Nutrient inputs from the compound fertilizer are calculated using the following Equation 1:

**Table 1:** Compound fertilizer inputs and organic amendment rates under different fertilization treatments.

Treatment	Compound fertilizer (kg ha^−1^)	N (kg ha^−1^)	P_2_O5 (kg ha^−1^)	K_2_O (kg ha^−1^)	Organic amendment	Rate (kg ha^−1^)	Note
CK	0	0	0	0	–	–	Control
CF	112	22.4	22.4	67.2	–	–	Compound fertilizer only
CFS	60.0	12.0	12.0	36.0	Corn straw	1530	Added to match total *N* deficit (Δ*N* = 10.4 kg N ha^−1^)
CFM	60.0	12.0	12.0	36.0	Pig manure	175	Added to match total *N* deficit (Δ*N* = 10.4 kg N ha^−1^)

CK, no fertilizer input (control); CF, compound fertilizer only. In CFS and CFM, the compound fertilizer rate was reduced relative to CF, and the resulting nominal total N deficit was supplemented with corn straw and pig manure, respectively. Application rates of the compound fertilizer and organic amendments were calculated from their measured nutrient contents as described in Section 2.2.This table summarizes compound fertilizer inputs and amendment rates, but does not imply equivalence in plant-available N, P, K, or C supply among treatments.

**Table 2:** Properties of corn straw and manure used for fertilization treatments in this study.

Properties	Corn straw	Manure
pH	8.47	6.3
Soil organic matter (g kg^−1^)	753.2	156.53
Total nitrogen (g kg^−1^)	6.8	59.43
Total phosphorus (g kg^−1^)	1.1	28.26
Total potassium (g kg^−1^)	14.4	32.23
Total carbon (g kg^−1^)	436.9	354.45

The analyses of the properties of corn straw and manure were conducted by Yunnan Sanbiao Agricultural and Forestry Technology Co., Ltd.


$$\begin{array}{l} {\text{N}}_{\text{comp}}={\;W}_{\text{N}}\times \text{R},\;{\text{P}}_{2}O_{5,comp}\;={\;W}_{{\text{P}}_{2}O_{5}}\times R,\;\\ \;{\text{K}}_{2}O_{\text{comp}}\;={\;W}_{{\text{K}}_{2}O}\times R \end{array}$$ (1)

Thus, with R = 112 kg ha^−1^ for CF, and R = 60 kg ha^−1^ for both CFS and CFM:

CF ( N: P_2_O_5_: K_2_O) = (22.4, 22.4, 67.2) kg ha^−1^, CFS and CFM ( N: P_2_O_5_: K_2_O) = (12.0, 12.0, 36.0) kg ha^−1^.

The N target for all fertilized treatments equals CF Equation 2:


$$\begin{array}{l} {\text{N}}_{\text{target}}=22.4\text{ kg }{\text{ha}}^{-1} \end{array}$$ (2)

Hence, the N deficit for CFS and CFM is Equation 3:


$$\begin{array}{l} \text{ΔN}={\text{N}}_{\text{target}}-{\text{N}}_{\text{comp}}\left(R=60\right)=\;22.4-12.0\;=10.4\text{ kg ha} \end{array}$$ (3)

Let *TN*_org_ be the total-N concentration (kg N kg^−1^ dry material). The required organic-amendment rate (kg ha^−1^, dry basis) is Equation 4:


$$\begin{array}{l} {\text{Rate}}_{\text{org}}=\;\frac{\text{Δ}N}{\text{T}{\text{N}}_{\text{org}}} \end{array}$$ (4)

Thus, for corn straw (6.8 g kg^−1^ = 0.0068 kg kg^−1^) and pig manure (59.43 g kg^−1^ = 0.05943 kg kg^−1^), using the following Equations 5, 6:


$$\begin{array}{l} {\text{Rate}}_{\text{straw}}\;=\frac{10.4}{0.0068}=1,529.41{\text{ kg ha}}^{-1}\approx 1,530{\text{ kg ha}}^{-1} \end{array}$$ (5)

$$\begin{array}{l} {\text{Rate}}_{\text{manure}}\;=\frac{10.4}{0.05943}=174.97{\text{ kg ha}}^{-1}\approx 175{\text{ kg ha}}^{-1} \end{array}$$ (6)

Accordingly, the manure was applied at 175 kg ha^−1^ in the CFM treatment, whereas corn straw was applied at 1,530 kg ha^−1^ in the CFS treatment. Both materials were collected from local farms and mixed into the topsoil by plowing in April. Flue-cured tobacco (*Nicotiana tabacum* L. “K326”) seedlings were transplanted in May and harvested in September. After harvesting, tobacco residues, such as stubble and roots, were completely removed to avoid interference with post-harvest soil measurements.

### 2.3 Soil sampling and analysis

Soil samples were collected from the same 12 plots at six key stages of flue-cured tobacco growth during the experiment conducted from May to September 2022. The six stages are: before transplanting (0 d), rosette stage (30 d), vigorous growth stage (45 d), topping stage (60 d), harvest stage (90 d), and post-curing stage (120 d). At each sampling date, one composite soil sample was collected from each plot by pooling five soil cores from the 0–20 cm layer using an aluminum cutting ring (5.4 cm high and 6 cm in diameter) following the methods described by (Wang et al. 2022a). Sampling positions within each plot were adjusted over time to avoid re-sampling previously disturbed locations. After removing visible plant residues, roots, gravel, and other debris, soil samples were sealed in polyethylene zipper bags and taken to the laboratory, then stored at 4 °C for subsequent analysis.

### 2.4 Determination of soil amino sugars

The determination of amino sugars was carried out according to the methods of (Hu et al. 2023). Briefly, 0.3 g of air-dried soil was weighed into a hydrolysis tube and mixed with 10 mL of 6 mol L^−1^ HCl. The mixture was hydrolyzed at 105 °C for 8 h. After cooling to room temperature, 100 μL of inositol solution (1 μg mL^−1^) was added as an internal standard (Internal Standard I), and the mixture was vortexed thoroughly. The hydrolysate was filtered through Whatman 2 Qualitative circles (125 mm diameter) to remove particulate residues, and the filtrate was evaporated to dryness using a rotary evaporator. The residue was dissolved in distilled water and the pH was adjusted to 6.6–6.8 by sequential addition of 1 mol L^−1^ KOH and 0.01 mol L^−1^ HCl. Samples were centrifuged at 1,600 × g for 10 min. The supernatant was collected and freeze-dried. The freeze-dried residue was washed three times with 1 mL methanol to remove residual polar impurities, and then derivatized to aldononitrile acetates. Derivatization was carried out by adding 300 μL of derivatization reagent to the freeze-dried sample and the mixture incubated at 80 °C for 30 min After cooling, 1 mL of acetic anhydride was added, and the sealed vial was heated again at 80 °C for 20 min. Upon cooling to room temperature, 1.5 mL of dichloromethane was added to extract the amino sugar derivatives. The excess acetic anhydride was removed by sequential washing the organic phase sequentially with 1 mL of 1 mol L^−1^ HCl (vortexed for 30 s, the aqueous layer was removed) followed by three washes with 1 mL of distilled water. The final organic phase was dried under nitrogen at 45 °C to remove dichloromethane. The residue was re-dissolved in 300 μL of an ethyl acetate:n-hexane mixture (v:v = 1:1) for final analysis.

### 2.5 Calculation of amino sugars

In this study, three amino sugars were observed: GluN, GalN and MurA. The concentrations (mg kg^−1^) of total amino sugars (Total Ass) were calculated by the sum of GluN, GalN, and MurA according to (Shao et al. 2017). Given that bacterial GluN and MurA are present in an approximate molar ratio of 2:1 (Li et al., 2015), the fungal-derived GluN fraction (FuGluN) was estimated by subtracting the bacterial contribution from total GluN content. The fungal-derived GluN is calculated using the following Equation 7 (Li et al., 2020a):


$$\begin{array}{l} FuGluN=GluN-2\times MurA\times 179.2/251.2 \end{array}$$ (7)

Fungal residue C (FRC, mg kg^−1^) and bacterial residue C (BRC, mg kg^−1^) were calculated based on the values of GluN and MurA, and microbial residue C (MRC, mg kg^−1^) was determined as the sum of FRC and BRC, by the following Equations 8–10, respectively (Luan et al., 2022):


$$\begin{array}{l} FRC=\left(GluN/179.2-2\times MurA/251.2\right)\times 179.2\times 9 \end{array}$$ (8)

$$\begin{array}{l} BRC=MurA\;\times \;45 \end{array}$$ (9)

$$\begin{array}{l} MRC=BRC\;+\;FRC \end{array}$$ (10)

Where:

251.2 and 179.2 are the molecular weight of MurA and GluN, respectively.

9 and 45 are the conversion factor of fungal GluN to fungal C (Engelking et al., 2007).

### 2.6 Quality assurance and quality control methods

Rigorous quality assurance and quality control procedures were implemented throughout the analytical process to ensure data accuracy and precision. All reagents were of chromatographic or guaranteed reagent grade, with ultrapure water (resistivity ≥18.2 MΩ·cm) used for all procedures. Method blanks (processed identically without soil) were analyzed with every 10 samples, demonstrating no detectable contamination of target amino sugars. Technical triplicates for each sample required ≤ 5% relative standard deviation for GluN, GalN, and MurA concentrations, samples exceeding this threshold were reanalyzed (Mou et al., 2020). Derivatization yield was verified using standard solutions (10 μg mL^−1^ each), requiring ≥90% conversion efficiency to aldononitrile acetates. Seven-point calibration curves (0.1–50 μg mL^−1^) exhibited linearity with R^2^ ≥0.997, recalibrated every 20 samples. GC-MS performance was validated daily with amino sugar reference standards, mandating ≤ 0.5% retention time deviation, signal-to-noise ratios ≥10, and ≥2,000 theoretical plates per peak (Maltari et al., 2026). Matrix-spiked quality control samples (10 μg mL^−1^) analyzed every 15 samples demonstrated acceptable recoveries of 80–94%. Raw data underwent outlier screening via Grubbs'test (α = 0.05), with exclusions limited to technically justifiable cases. Results were reported as mean ± SD of triplicates, with significant figures aligned to analytical precision. Certified reference materials were analyzed periodically to verify method accuracy, with relative errors maintained within ± 5%. Based on 10 blank replicates, the method detection limit and method quantification limit were 0.10 and 0.30 mg kg^−1^, respectively, for both GluN and GalN, and 0.01 and 0.03 mg kg^−1^, respectively, for MurA, indicating adequate sensitivity for trace-level determination in soil samples.

### 2.7 Statistical analysis

All statistical analyses and figures preparation were performed using in Python 3.13.12 (packages: pandas 3.0.1, SciPy 1.17.1, statsmodels 0.14.6, matplotlib 3.10.8, and openpyxl 3.1.5). Data are presented as mean ± standard error (SE) of three field replicates. Differences among treatments within the same sampling time and among sampling times within the same treatment were analyzed by one-way analysis of variance followed by Duncan's multiple range test (Long et al., 2025), and significance was accepted at P < 0.05. Fertilization treatment, sampling time, and their interaction were further evaluated using linear mixed-effects models (Huang et al., 2022), with treatment, time, and treatment × time as fixed effects and block and plot structure incorporated to account for the randomized block design and repeated observations over time. The significance of fixed effects was assessed by likelihood ratio tests comparing nested models fitted by maximum likelihood. Pearson correlation analysis was used to examine relationships among SOC, amino sugar variables, microbial residue carbon fractions, and soil physicochemical properties. To further evaluate the relationships among fertilization treatment, sampling time, soil physicochemical properties, microbial residues, and SOC, a merged piecewise linear mixed-effects structural equation model was fitted (Lefcheck, 2016; Huang et al., 2022). Soil physicochemical properties and microbial residues were represented by the first principal components of the corresponding standardized variables (Jolliffe and Cadima, 2016). C/N and derived residue variables were excluded to avoid circularity and redundancy. Model fit was assessed using Shipley's test of d-separation and Fisher's C statistics.

## 3 Results

### 3.1 Effects of fertilization regimes on tobacco soil nutrients and soil organic carbon

As shown in Table 3, fertilization regime significantly affected soil pH and nutrient dynamics during the tobacco-growing period. In all fertilized treatments, soil pH declined during the early stage and reached minima at 30 d, with values of 6.08 ± 0.01 under CFM, 5.94 ± 0.02 under CFS, and 5.57 ± 0.01 under CF, whereas CK remained higher at 7.47 ± 0.01. Thereafter, pH gradually recovered. TN showed a similar treatment-dependent pattern. CF reached the highest TN at 30 d (1.50 ± 0.01 g kg^−1^), but declined to 0.58 ± 0.01 g kg^−1^ at 120 d, whereas CFM maintained a comparatively higher late-season TN level (0.80 ± 0.04 g kg^−1^). TP also peaked at 30 d under CF and CFM (1.67 ± 0.02 and 1.61 ± 0.02 g kg^−1^, respectively), but remained higher under CFM (1.16 ± 0.02 g kg^−1^) than under CF (1.07 ± 0.02 g kg^−1^) at 120 d. ${\text{NH}}_{4}^{+}$ and NO3^−^ concentrations were highly dynamic over time. At 45 d, ${\text{NH}}_{4}^{+}$ was highest under CF (6.56 ± 0.06 mg kg^−1^), whereas NO3^−^ reached maximum values under CFM (40.84 ± 0.42 mg kg^−1^) and CF (38.54 ± 0.50 mg kg^−1^). The C/N ratio also varied considerably, ranging from 4.65 ± 0.13 under CF at 30 d to 16.46 ± 0.47 under CFM at 45 d, indicating clear treatment-specific differences in carbon and nitrogen balance.

**Table 3:** Physicochemical properties of tobacco-planting soil under different treatments at various growth stages.

Time (day)	Treatment	pH	Total nitrogen (g kg^−1^)	Total phosphorus (g kg^−1^)	Ammonium nitrogen (mg kg^−1^)	Nitrate nitrogen (mg kg^−1^)	C/N
0d	CFM	7.07 ± 0.01Dd	0.95 ± 0.02Ba	1.34 ± 0.01Ca	2.81 ± 0.07Aa	27.71 ± 0.57Ba	9.50 ± 0.19Cb
	CFS	7.23 ± 0.01Ab	0.75 ± 0.02Bb	1.18 ± 0.01Cb	1.15 ± 0.00Cc	27.56 ± 0.55Aa	9.44 ± 0.09BCb
	CF	7.16 ± 0.01Cc	0.76 ± 0.02Bb	1.07 ± 0.02Cc	1.94 ± 0.07Cb	18.63 ± 0.42Cb	9.50 ± 0.39Db
	CK	7.34 ± 0.02Da	0.59 ± 0.01Bc	1.00 ± 0.01ABd	0.23 ± 0.04Ad	14.12 ± 0.42Bc	11.53 ± 0.50ABa
30d	CFM	6.08 ± 0.01Eb	1.32 ± 0.01Ab	1.61 ± 0.02Ab	1.23 ± 0.00Bc	19.40 ± 0.02Cc	7.78 ± 0.13Db
	CFS	5.94 ± 0.02Dc	1.11 ± 0.03Ac	1.44 ± 0.02Ac	2.26 ± 0.00Ba	27.84 ± 0.04Aa	7.23 ± 0.31Db
	CF	5.57 ± 0.01Ed	1.50 ± 0.01Aa	1.67 ± 0.02Aa	2.11 ± 0.00Bb	23.73 ± 0.04Bb	4.65 ± 0.13Ec
	CK	7.47 ± 0.01Ca	0.74 ± 0.02Ad	1.02 ± 0.01Ad	0.22 ± 0.02Ad	27.85 ± 0.28Aa	10.17 ± 0.39Ba
45d	CFM	7.03 ± 0.01Db	0.58 ± 0.01Ec	1.42 ± 0.02Ba	0.25 ± 0.00Dc	40.84 ± 0.42Aa	16.46 ± 0.47Aa
	CFS	6.98 ± 0.01Cc	0.77 ± 0.02Ba	1.24 ± 0.02Bb	3.36 ± 0.09Ab	12.65 ± 0.25Cc	9.36 ± 0.31Cc
	CF	6.79 ± 0.01Dd	0.67 ± 0.01Cb	1.13 ± 0.01Bc	6.56 ± 0.06Aa	38.54 ± 0.50Ab	10.12 ± 0.42CDc
	CK	7.48 ± 0.01Ca	0.57 ± 0.03Bc	1.02 ± 0.01Ad	0.16 ± 0.03Ac	12.82 ± 0.42Cc	12.79 ± 0.76Ab
60d	CFM	7.31 ± 0.02Ac	0.70 ± 0.01Da	1.29 ± 0.01Da	1.05 ± 0.10Cb	12.60 ± 0.40Da	12.38 ± 0.38Ba
	CFS	7.14 ± 0.01Bd	0.65 ± 0.02Cb	1.12 ± 0.01Db	0.93 ± 0.07Db	8.59 ± 0.11Db	11.55 ± 0.59Aa
	CF	7.49 ± 0.01Ab	0.69 ± 0.01Ca	1.15 ± 0.02Bb	1.72 ± 0.10Da	9.20 ± 0.15Db	11.61 ± 0.30ABa
	CK	7.77 ± 0.01Aa	0.59 ± 0.01Bc	1.01 ± 0.01Ac	0.19 ± 0.01Ac	4.02 ± 0.27Ec	11.69 ± 0.57ABa
90d	CFM	7.26 ± 0.01Bb	0.67 ± 0.03Da	1.19 ± 0.01Ea	0.33 ± 0.01Dab	5.91 ± 0.36Fb	11.72 ± 0.29Ba
	CFS	7.15 ± 0.01Bc	0.66 ± 0.01Ca	1.10 ± 0.01Db	0.36 ± 0.02Fa	14.56 ± 0.23Ba	10.64 ± 0.44ABa
	CF	7.25 ± 0.01Bb	0.57 ± 0.01Db	1.04 ± 0.02Cc	0.27 ± 0.02Eb	4.80 ± 0.15Fc	12.74 ± 0.60Aa
	CK	7.61 ± 0.01Ba	0.62 ± 0.02Bab	0.98 ± 0.01Bd	0.14 ± 0.02Ac	3.95 ± 0.09Ed	11.15 ± 0.61ABa
120d	CFM	7.18 ± 0.01Cb	0.80 ± 0.04Ca	1.16 ± 0.02Ea	0.30 ± 0.01Db	8.01 ± 0.34Ea	12.13 ± 0.58Ba
	CFS	7.21 ± 0.01Ab	0.68 ± 0.01Cb	0.98 ± 0.02Ec	0.70 ± 0.10Ea	6.32 ± 0.45Eb	10.79 ± 0.42Aa
	CF	7.21 ± 0.02Bb	0.58 ± 0.01Dc	1.07 ± 0.02Cb	0.25 ± 0.02Eb	6.29 ± 0.30Eb	11.09 ± 0.37BCa
	CK	7.46 ± 0.01Ca	0.51 ± 0.01Cc	0.85 ± 0.01Cd	0.15 ± 0.02Ab	6.07 ± 0.22Db	12.73 ± 0.42Aa
Mixed-effects model with block and plot (LR χ^2^ value)				
Treatment		34.910^***^	25.559^***^	42.272^***^	24.586^***^	14.124^**^	18.700^***^
Time		96.029^***^	98.857^***^	95.490^***^	35.525^***^	87.029^***^	70.280^***^
Treatment × Time		419.595^***^	248.816^***^	198.137^***^	401.263^***^	437.110^***^	177.847^***^

Data are presented as mean ± standard error (SE). Different lowercase letters within the same sampling time indicate significant differences among fertilization treatments according to Duncan's multiple range test (*P* < 0.05), whereas different uppercase letters within the same fertilization treatment indicate significant differences among sampling times (*P* < 0.05). The overall effects of fertilization treatment, sampling time, and their interaction were evaluated using linear mixed-effects models accounting for block and plot structure. Values in the bottom three rows are likelihood-ratio chi-square statistics from the mixed-effects models. Asterisks indicate significance levels: ^**^P < 0.01, and ^***^P < 0.001.

**Figure 1:**
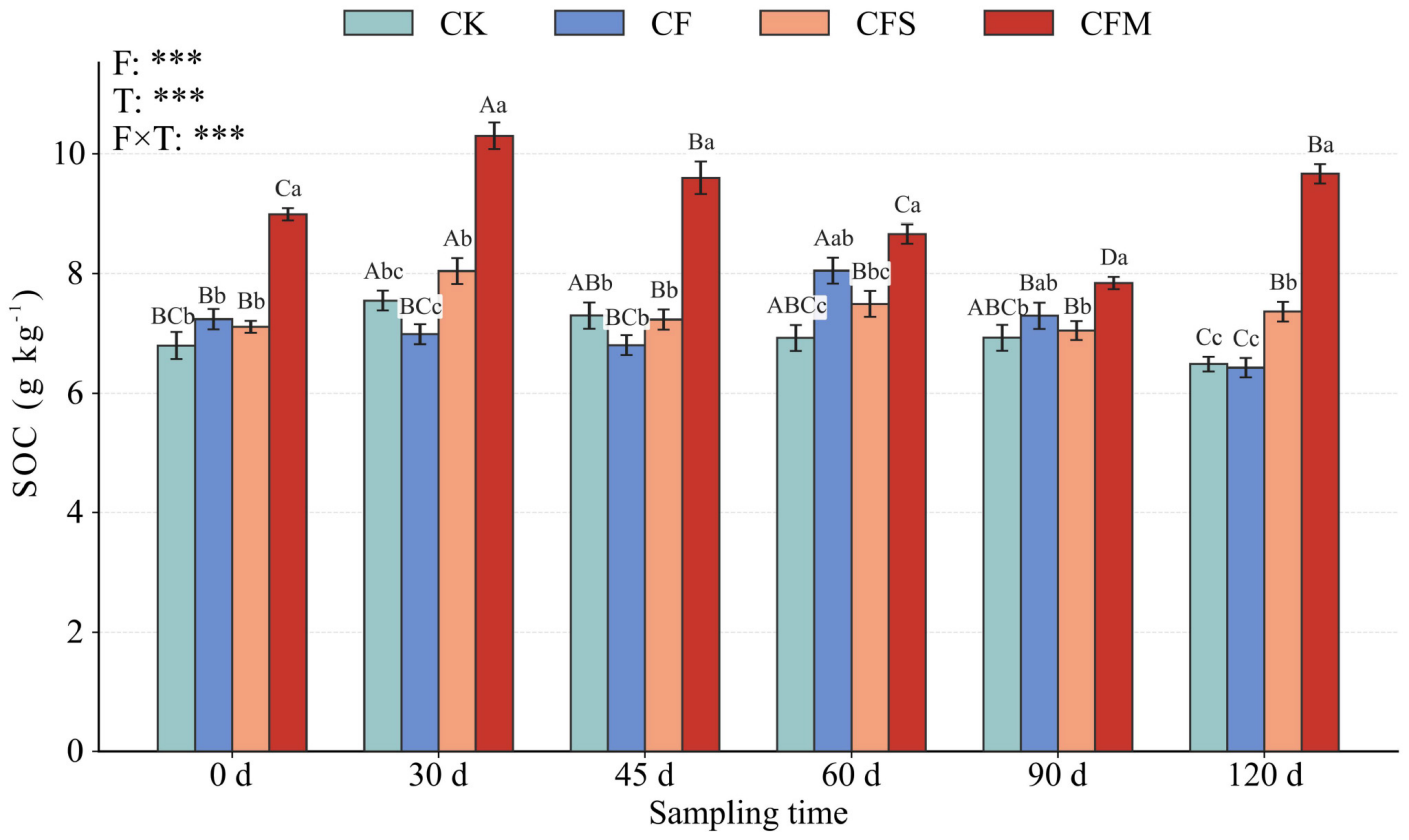
Effects of different fertilization treatments on soil organic carbon (SOC) content. Bars represent mean ± standard error (SE). Different lowercase letters indicate significant differences among fertilization treatments within the same sampling time, whereas different uppercase letters indicate significant differences among sampling times within the same fertilization treatment according to Duncan's multiple range test at P < 0.05. F (Treatment), T (Time), and F × T denote the overall effects of fertilization treatment, sampling time, and their interaction, respectively, as evaluated by linear mixed-effects models accounting for block and plot structure. Asterisks indicate significance levels: ***P < 0.001.

The temporal pattern of SOC concentration generally followed the variation in soil nutrient status (Figure 1). CFM consistently showed the highest SOC concentration, increasing from 8.99 ± 0.10 g kg^−1^ at 0 d to 10.30 ± 0.22 g kg^−1^ at 30 d, declining to 7.84 ± 0.10 g kg^−1^ at 90 d, and recovering to 9.66 ± 0.16 g kg^−1^ at 120 d. CFS showed an intermediate response, with SOC increasing from 7.11 ± 0.10 g kg^−1^ at 0 d to 8.04 ± 0.22 g kg^−1^ at 30 d and at 7.36 ± 0.16 g kg^−1^ at 120 d. In contrast, CF showed only a transient increase, reaching 8.05 ± 0.22 g kg^−1^ at 60 d before declining to 6.43 ± 0.16 g kg^−1^ at 120 d, whereas CK remained comparatively low throughout the season, ranging from 6.49 ± 0.12 to 7.55 ± 0.17 g kg^−1^. Linear mixed-effects models further confirmed significant effects of fertilization treatment, sampling time, and their interaction on soil physicochemical properties and SOC concentration, indicating that fertilization regime and temporal dynamics jointly shaped short-term changes in nutrient status and SOC in tobacco-planted soil.

### 3.2 Effects of fertilization regimes on soil amino sugars and microbial residues

**Figure 2:**
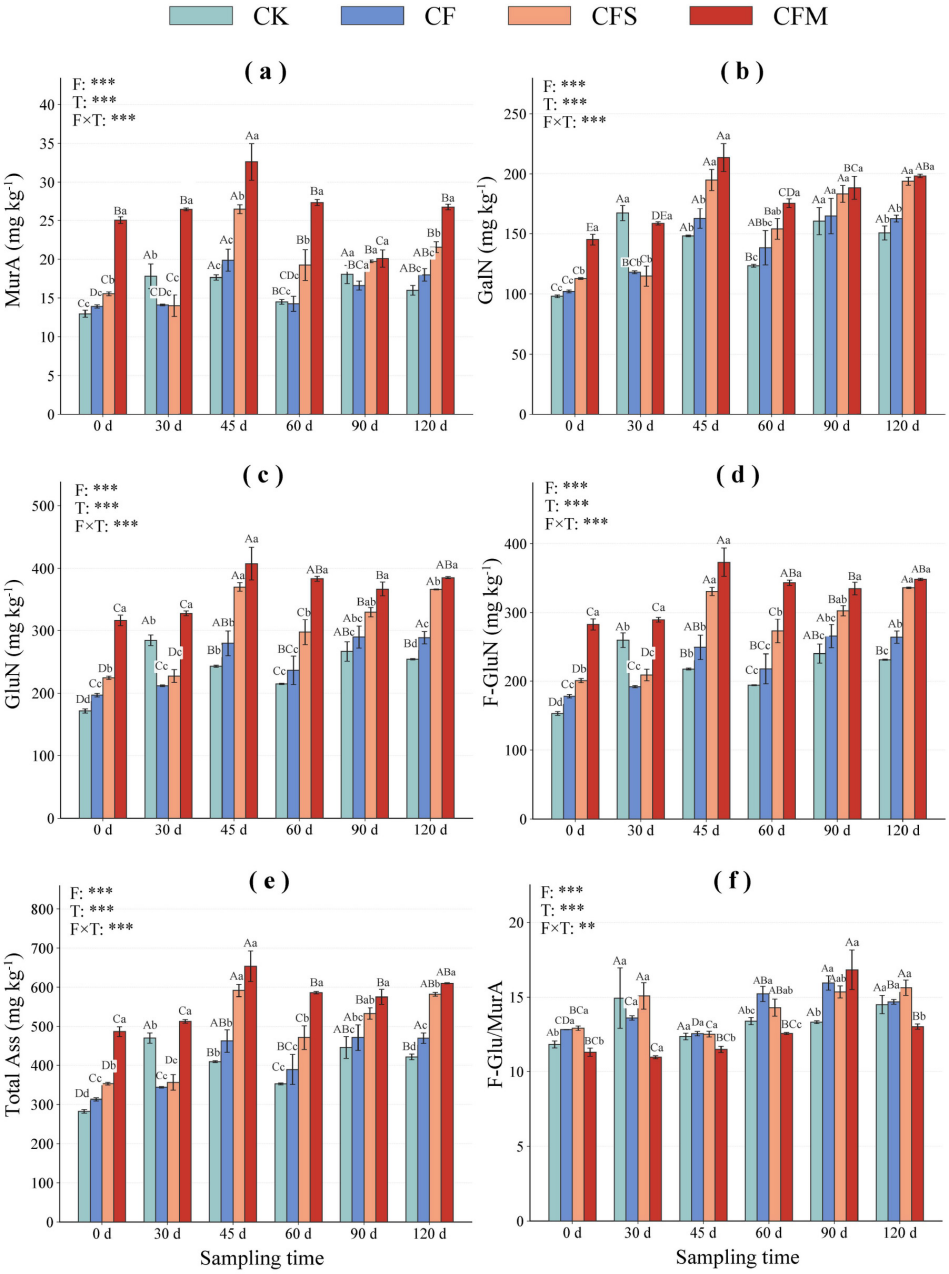
Temporal changes in amino sugar biomarkers under different fertilization treatments during the tobacco-growing. **(a–f)** show muramic acid (MurA), galactosamine (GalN), glucosamine (GluN), fungal glucosamine (F-GluN), total amino sugars (Total Ass), and the F-GluN/MurA ratio, respectively. Bars represent mean ± SE. Asterisks indicate significance levels: **P < 0.01, and ***P < 0.001.

Fertilization significantly altered amino sugar concentrations over time (Figure 2), and the overall treatment pattern during the middle and late stages was generally CFM > CFS > CF > CK. MurA under CFM remained higher than under the other treatments during 0–60 d and again at 120 d. At 30 d, CK showed relatively high values of GalN (167.29 mg kg^−1^), GluN (284.23 mg kg^−1^), F-GluN (259.39 mg kg^−1^), and total amino sugar (469.31 mg kg^−1^), whereas by 45 d these variables peaked under CFM, with GluN, F-GluN, and Total Ass reaching 407.34, 373.09, and 653.70 mg kg^−1^, respectively. After 45 d, CFM generally maintained higher amino sugar concentrations than the other treatments. The F-GluN/MurA ratio also varied over time, reaching 10.76 at 30 d and 18.88 at 90 d under CFM.

**Figure 3:**
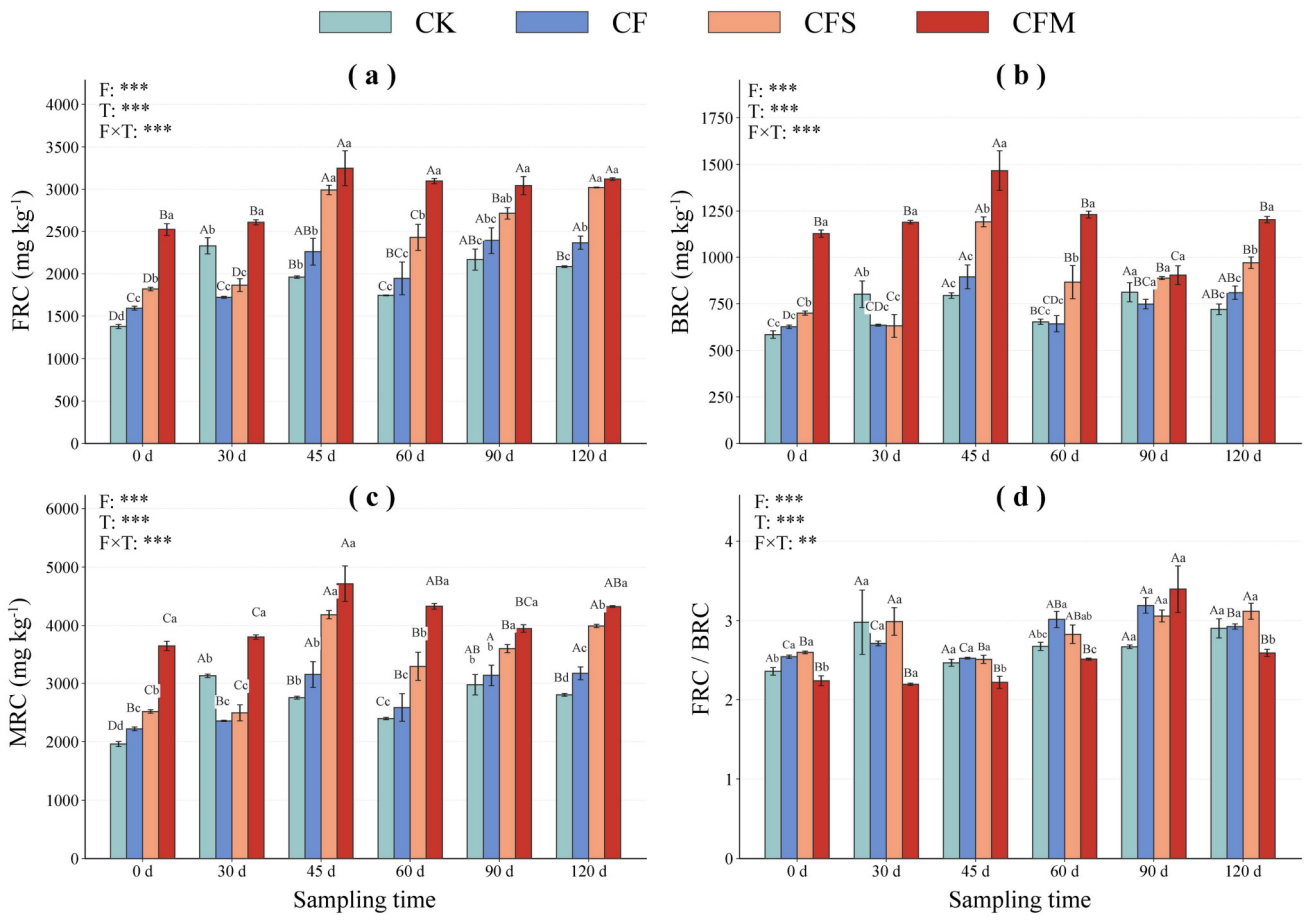
Temporal changes in microbial residue carbon fractions under different fertilization treatments during the tobacco-growing. **(a–d)** show fungal residue carbon (FRC), bacterial residue carbon (BRC), microbial residue carbon (MRC), and the FRC/BRC ratio, respectively. Asterisks indicate significance levels: **P < 0.01, and ***P < 0.001.

Microbial residue carbon fractions also differed among treatments and sampling times (Figure 3). At 45 d, FRC under CFM reached 3,248.34 mg kg^−1^, compared with 2,993.03, 2,259.96, and 1,960.20 mg kg^−1^ under CFS, CF, and CK, respectively. At the same stage, BRC was highest under CFM (1,467.45 mg kg^−1^), exceeding CFS (1,191.30 mg kg^−1^), CF (894.15 mg kg^−1^), and CK (793.95 mg kg^−1^). Total MRC peaked under CFM (4,715.79 mg kg^−1^ at 45 d) representing a 71.2 % increase over CK (2,754.15 mg kg^−1^), with CFS and CF showing +51.9 % (4,184.33 mg kg^−1^) and +14.5 % (3,154.11 mg kg^−1^) respectively.

### 3.3 Effects of soil microbial residues on SOC

**Figure 4:**
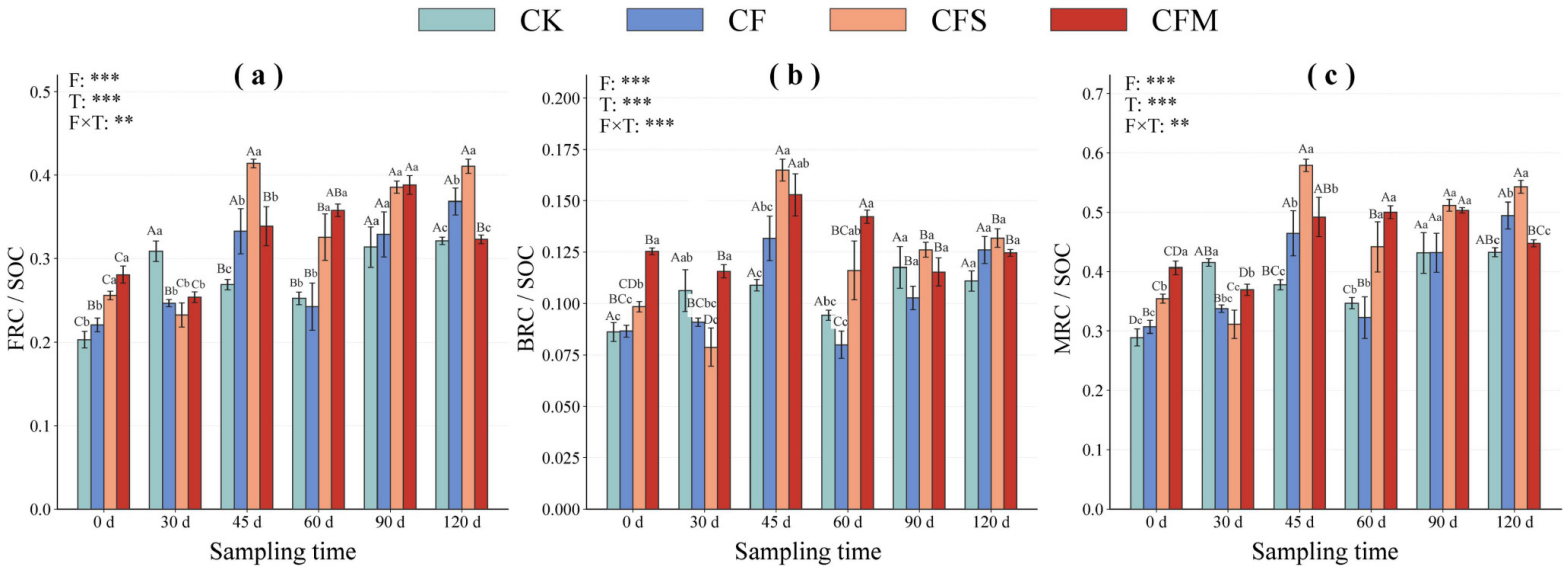
Temporal changes in the contributions of microbial residue carbon fractions to SOC under different fertilization treatments during the tobacco-growing season. **(a–c)** show FRC/SOC, BRC/SOC, and MRC/SOC, respectively. Asterisks indicate significance levels: **P < 0.01, and ***P < 0.001.

Soil microbial residues contributed differently to SOC under various fertilization regimes during the flue-cured tobacco growing season (Figure 4). Under CFS, FRC/SOC reached 41.41% at 45 d, which was 53.9% higher than CK. Meanwhile CFM initially indicated lower FRC/SOC by 17.8 % after 30 d cultivation compared to CK. The insignificant reduction (25.38 % vs. 30.88 %), then rose up to 35.8 % of FRC/SOC at 60 d (+41.8 % vs. CK). BRC/SOC under CFS reached a maximum peak at 16.5 % on 45 d which was 51.5 % higher than CK, whereas both CFS and CFM generally maintained higher BRC/SOC than CK and CF during much of the growing season. Under CFM, MRC/SOC increased by 30.2–44.3 % during 45–60 d, but declined after 90 d. These results indicate that organic amendments altered both the magnitude and the temporal pattern of residue-C contributions to SOC.

### 3.4 Correlation and SEM analysis

**Figure 5:**
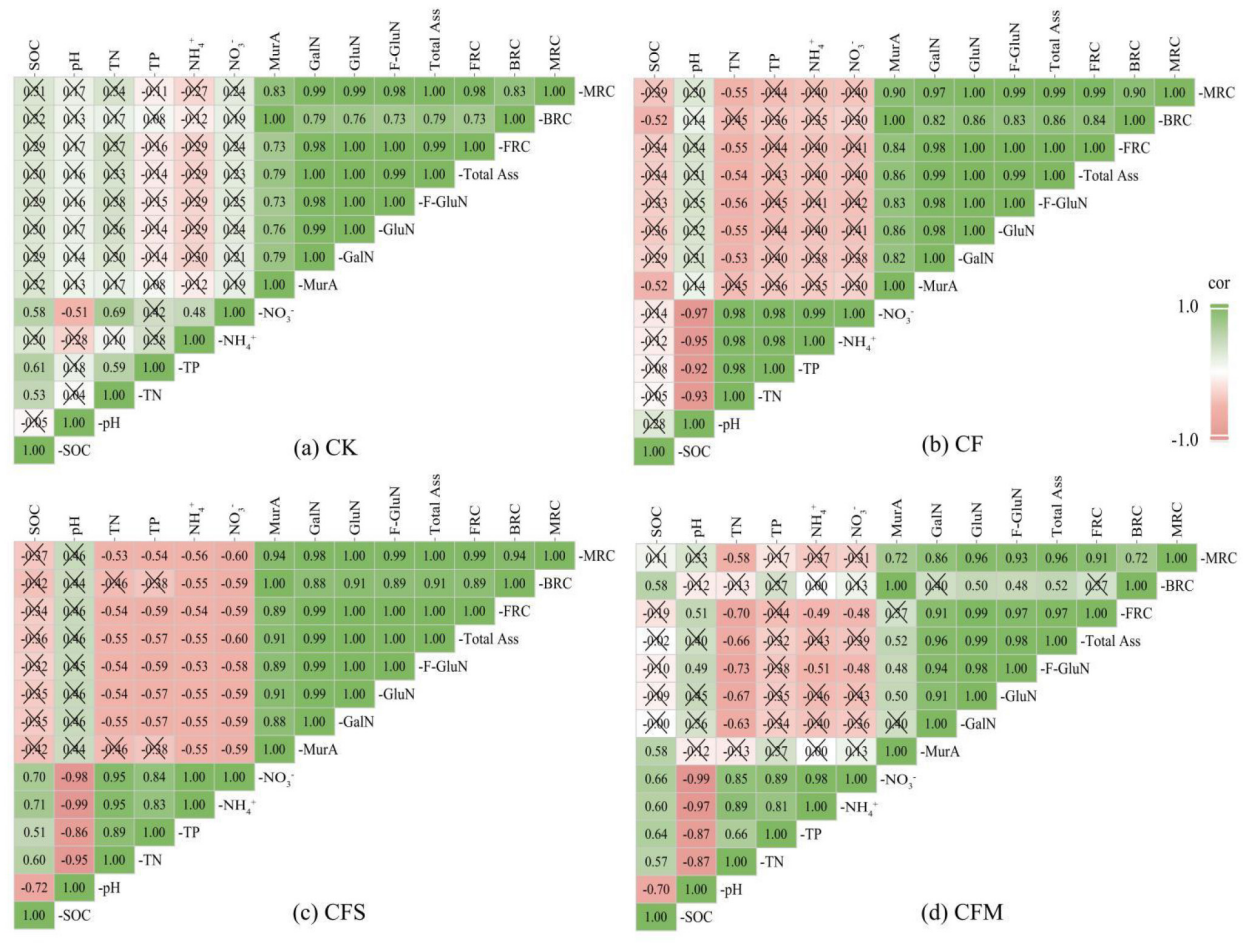
Correlation analysis between SOC, soil nutrients, and microbial necromass under different fertilization treatments: **(a)** CK, **(b)** CF, **(c)** CFS, and **(d)** CFM. Heatmaps of pairwise Pearson correlations among soil physicochemical properties and amino sugar–derived pools under different fertilization treatments. Numbers in the cells indicate the Pearson correlation coefficients (r). Cell color denotes the strength of the correlation (green = positive, red = negative). Statistical significance is indicated as follows: cells marked with “ × ” represent non-significant correlations (*P* ≥ 0.05), whereas cells with numeric entries correspond to significant correlations (*P* < 0.05).

**Figure 6:**
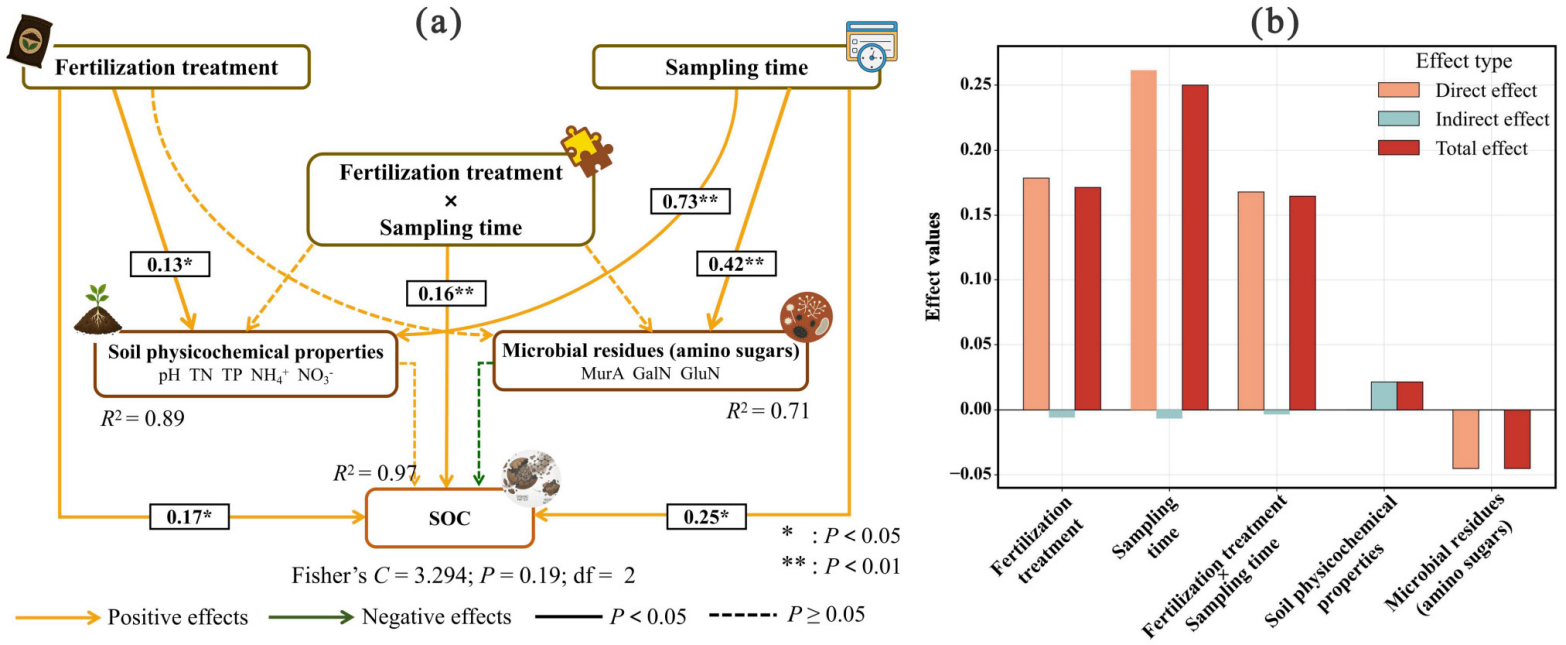
Structural equation model showing the effects of fertilization treatment, sampling time, and their interaction on soil physicochemical properties, microbial residues, and SOC **(a)**, together with the direct, indirect, and total effects derived from the same model **(b)**. Solid and dashed lines indicate significant (*P* < 0.05) and non-significant (*P* ≥ 0.05) relationships, respectively. Values adjacent to arrows indicate effect values for fixed factors or standardized path coefficients for continuous paths, and only significant pathways are labeled. *R*^2^ indicates the proportion of variance explained for each endogenous variable. Model fit was assessed using Shipley's test of d-separation and Fisher's C statistic, and the SEM showed acceptable fit (Fisher's C = 3.294, df = 2, *P* = 0.19).

Pearson correlation analysis showed that the relationships between SOC and soil or residue variables differed among fertilization regimes (Figure 5). SOC exhibited a significant negative correlation with soil pH under CFS and CFM (*r* = −0.70 to −0.72, *P* < 0.05), whereas no significant correlation was observed under CK and CF. In CFS and CFM, SOC was also positively correlated with TN, TP, NH^4+^, NO^3−^ (all *r* > 0.5, *P* < 0.05). At the same time, SOC was positively correlated with MurA (r = 0.58, *P* < 0.05) under CFM. The merged piecewise linear mixed-effects SEM showed acceptable fit (Fisher's C = 3.294, df = 2, *P* = 0.193) (Figure 6). Fertilization treatment, sampling time, and their interaction all had significant positive effects on soil physicochemical properties, microbial residues, and SOC, with effect values of 0.13, 0.73, and 0.13 for soil physicochemical properties, 0.11, 0.42, and 0.13 for microbial residues, and 0.17, 0.25, and 0.17 for SOC, respectively (all *P* < 0.001). Effect decomposition further showed that sampling time had the strongest total effect (0.24), followed by fertilization treatment (0.17) and treatment × time (0.16). Overall, short-term SOC dynamics were driven mainly by fertilization regime and temporal variation.

## 4 Discussion

### 4.1 Mechanisms driving short-term SOC accumulation under fertilization regimes

SOC is closely linked to a wide range of soil physicochemical and biological properties and processes, and is widely recognized as a key indicator of soil functioning, fertility, and health (Koorneef et al., 2024). In our study, SOC concentration was consistently higher under the two organic-amended treatments than under CF and CK, and CFM generally showed the largest short-term response during the tobacco-growing period (Figure 1). This pattern was accompanied by more sustained late-season TN and TP under CFM, whereas CF showed a sharper decline after the early nutrient peak (Table 3). These results suggest that short-term SOC accumulation in this tobacco soil was associated not only with external organic C input, but also with a more favorable nutrient context for C retention. Similar patterns have been reported in other agricultural soils, where the combined application of mineral fertilizer and organic amendments increased SOC relative to mineral fertilization alone by improving soil nutrient status and promoting the retention of amendment-derived C in soil (Bai et al., 2019; Dai et al., 2021; Chen et al., 2022; Shen et al., 2022). A likely explanation is that short-term SOC accumulation under fertilization is jointly regulated by substrate supply and the soil environment in which that substrate is processed. Our study showed that the temporal pattern of SOC generally followed the variation in soil nutrient status, and the organic-amended treatments also showed stronger changes in pH and mineral N dynamics than CF and CK. Notably, based on the measured compositions of the amendments, straw contributed relatively more carbon and potassium, whereas manure contributed relatively more phosphorus and a different form of organic substrate input in this study. This difference likely contributed to the distinct nutrient trajectories and SOC responses observed under the two treatments. Accordingly, it indicates that, under this short-term tobacco field condition, the manure-associated resource package was more favorable for SOC retention. This interpretation is broadly consistent with studies showing that different organic amendments regulate SOC dynamics differently depending on their chemical composition and the way they interact with soil microbial processing and stabilization pathways (Cotrufo and Lavallee, 2022). Previous studies in tobacco-planting soils have also mainly shown that fertilization and organic amendments can alleviate soil acidification, improve nutrient availability, and increase SOC or related carbon pools (Bai et al., 2019; Dai et al., 2021; Chen et al., 2022; Shen et al., 2022). However, these studies have generally focused on physicochemical properties, microbial community composition, or overall carbon responses, and have rarely resolved how fungal- and bacterial-derived residues differentially contribute to SOC formation in tobacco soils. Overall, our results further support the positive role of organic amendments in short-term SOC enrichment in tobacco-planted soil and highlight the importance of amendment-derived resource environments in shaping SOC responses.

### 4.2 Amino sugar-derived residue patterns and their associations with SOC

In this study, our results showed that amino sugar concentrations (Figure 2) and microbial residue-C fractions (Figure 3) were generally higher in the two organic-amended treatments than in CF and CK, especially during the middle and late stages. In particular, CFM maintained higher MurA during most of the observation period and showed the highest values of GalN, GluN, F-GluN, Total Ass, FRC, BRC, and MRC at 45 d (Figures 2, 3). These results suggest that manure amendment created a more favorable environment for the accumulation of microbial residue-related compounds. And CFS exhibited a relatively higher FRC/SOC ratio at some stages, whereas CFM maintained consistently higher BRC content and BRC/SOC ratio during much of the observation period (Figure 4). This suggests that straw and manure amendments were associated with different modes of microbial residue allocation within the SOC pool. Some studies showed that organic amendments enhance amino sugar accumulation and microbial necromass-related C pools relative to mineral fertilization alone (Liang et al., 2020; Ni et al., 2020; Li et al., 2024). Taken together, our results suggest that organic-amended fertilization influenced amino sugar-derived residue patterns linked to SOC, but the biological processes underlying these patterns require further verification using direct measurements of living microbial activity and function.

### 4.3 Associations among soil physicochemical properties, microbial residues, and SOC

The correlation and SEM results further suggest that short-term SOC dynamics in this tobacco soil were jointly shaped by fertilization regime and temporal variation (Figures 5, 6). Fertilization-induced environmental shifts (e.g., soil acidification and stoichiometric imbalance) created synergistic interactions between soil nutrient and SOC accumulation (Table 3). In our study, SOC showed a significant negative correlation with soil pH, and positively correlated with TN, TP, $NH_{4}^{+}$, and NO3^−^ in the two organic-amended treatments, indicating that short-term SOC accumulation occurred together with marked shifts in soil acidity and nutrient availability. Similar study showed that under acidic conditions, the decomposition rate of organic matter decreases, while the increased negative charge on iron and aluminum oxides promotes the formation of organo-mineral complexes (Kleber et al., 2021). What's more, our findings showed that MurA exhibit a positive correlation with SOC content. This suggested such as peptidoglycan, significantly increase the bacterial residues during the late growth phase due to their resistance to decomposition (Miltner et al., 2012). In addition, the initial soil contained 22% clay, which may have provided mineral surfaces for the adsorption and protection of bacterial residues. Similarly, (Simpson et al. 2007) pointed out that bacterial cell wall components can form stable organic-mineral complexes through mineral binding or physical encapsulation. The piecewise linear mixed-effects SEM, which showed that fertilization treatment, sampling time, and their interaction were the dominant drivers of short-term SOC dynamics, with sampling time having the strongest total effect. Thus, unlike studies that infer strong direct residue-mediated control over SOC stabilization, our results suggest that short-term SOC variation in this system was driven mainly by fertilization regime and temporal dynamics, while residue indicators reflected part of the associated soil response.

While this study elucidated the short-term effects of microbial residue dynamics on SOC under different fertilization regimes, the limited observation period of 120 d may not fully capture the long-term dynamics and persistence of the SOC under fertilization (Poeplau and Don, 2015). In addition, as a single-site study conducted in Yunnan Province, the present findings are primarily applicable to tobacco-planting systems with similar red-soil conditions, continuous-cropping history, and fertilization backgrounds. Although routine management of the target plot did not involve intentional large-scale organic amendments apart from the documented historical trial seasons, potential legacy effects from previous field activities cannot be fully excluded and should be considered. Furthermore, the absence of direct measurements of microbial respiration, extracellular enzyme activities, and functional genes limits our ability to resolve the physiological and molecular mechanisms underlying residue formation and SOC accumulation (Trivedi et al., 2016). The lack of stable isotope tracing is another limitation, as it prevented direct quantification of the relative contributions of bacterial and fungal residues to SOC formation. Overall, our findings support that different fertilization regimes, particularly those including organic amendments, were associated with distinct combinations of soil properties, amino sugar-derived residue signatures, and SOC responses, but the mechanistic pathways underlying these linkages remain to be clarified. Future studies should incorporate long-term multi-site field experiments with isotope tracing and multi-omics approaches to better clarify the temporal persistence, regional applicability, and mechanistic basis of microbial residue-mediated SOC stabilization under different fertilization strategies.

## 5 Conclusions

This study demonstrates that the combined application of chemical fertilizer with organic amendments favored short-term SOC accumulation in continuously cropped tobacco-planted red soil. Compared with CF and CK, the two organic-amended treatments were associated with higher SOC concentrations, with CFM showing the strongest response. Organic-amended fertilization also increased amino sugar concentrations and microbial residue-C fractions, and BRC and MurA were more closely associated with SOC than FRC during the observed period. The merged piecewise linear mixed-effects SEM indicated that short-term SOC dynamics were driven mainly by fertilization regime and temporal variation. Overall, the results indicate that fertilization regime and temporal variation jointly shaped short-term SOC dynamics in this system, and these findings provide region-specific evidence for optimizing fertilization strategies in tobacco-planting soils of Yunnan.

## Data Availability

The original contributions presented in the study are included in the article/supplementary material, further inquiries can be directed to the corresponding authors.
